# Nutritional Signaling via Free Fatty Acid Receptors

**DOI:** 10.3390/ijms17040450

**Published:** 2016-03-25

**Authors:** Junki Miyamoto, Sae Hasegawa, Mayu Kasubuchi, Atsuhiko Ichimura, Akira Nakajima, Ikuo Kimura

**Affiliations:** 1 Department of Applied Biological Science, Graduate School of Agriculture, Tokyo University of Agriculture and Technology, Tokyo 183-0057, Japan; d142212@hiroshima-u.ac.jp (J.M.); 50012152057@st.tuat.ac.jp (S.H.); 50012152019@st.tuat.ac.jp (M.K.); anakajima@ak.med.kyoto-u.ac.jp (A.N.); 2 Department of Biological Chemistry, Graduate School of Pharmaceutical Science, Kyoto University, Kyoto 606-8501, Japan; ichimura.atsuhiko.2r@kyoto-u.ac.jp

**Keywords:** FFAR, fatty acids, GPR120, GPR41, GPR43, GPR40

## Abstract

Excess energy is stored primarily as triglycerides, which are mobilized when demand for energy arises. Dysfunction of energy balance by excess food intake leads to metabolic diseases, such as obesity and diabetes. Free fatty acids (FFAs) provided by dietary fat are not only important nutrients, but also contribute key physiological functions via FFA receptor (FFAR)-mediated signaling molecules, which depend on FFAs’ carbon chain length and the ligand specificity of the receptors. Functional analyses have revealed that FFARs are critical for metabolic functions, such as peptide hormone secretion and inflammation, and contribute to energy homeostasis. In particular, recent studies have shown that the administration of selective agonists of G protein-coupled receptor (GPR) 40 and GPR120 improved glucose metabolism and systemic metabolic disorders. Furthermore, the anti-inflammation and energy metabolism effects of short chain FAs have been linked to the activation of GPR41 and GPR43. In this review, we summarize recent progress in research on FFAs and their physiological roles in the regulation of energy metabolism.

## 1. Introduction

Although diet is the most important source of daily nutrients, excess food intake leads to metabolic diseases, such as type 2 diabetes (T2D) and obesity. In recent years, obesity is one of the most critical health problems worldwide [1,2]. In the past decades, it has become increasingly apparent that all macronutrients, including carbohydrates, proteins and lipids, play an important role in the regulation of energy metabolism. Obesity develops as a result of a prolonged imbalance between energy expenditure and intake, which influences various metabolite- and hormone-related pathways [3]. Excessive food intake along with insufficient exercise and genetic susceptibility is considered a risk factor for obesity. Recent studies also suggest that the intestinal environment, particularly changes in gut microbiota composition, is closely linked to obesity and metabolic disorders [4,5,6]. One role of gut microbiota is to exchange free fatty acids (FFAs) derived from dietary fat for other FFAs as gut microbial metabolites that are key factors in energy metabolism.

FFAs are not only an essential energy source, but also function as signaling molecules that regulate various cellular processes and physiological functions according to carbon chain length. Short chain fatty acids (SCFAs) have no more than six carbons; medium chain fatty acids (MCFAs) have from 6–12 carbons; and long chain fatty acids (LCFAs) have greater than 12 carbons. Humans are unable to generate linoleic acid C18:2 (ω-6) and α-linolenic acid C18:3 (ω-3), because humans cannot synthesize the ω-6 or ω-3 double bond. Therefore, these fatty acids must be obtained from the diet. Humans can elongate and desaturate linoleic and α-linolenic acids to longer chain products, with variable rates of efficiency. MC- and LCFAs are metabolized by β-oxidation and are provided in tissues as an energy source. Dietary fibers have had interest in them owing to their beneficial metabolic effects on glucose homeostasis, food intake, insulin sensitivity and body weight [7,8]. SCFAs, such as acetate, propionate and butyrate, are produced by gut microbial fermentation of dietary fibers [9]. Gut microbiota affect host nutrient acquisition and energy regulation and can influence the development of obesity and diabetes [10]. Dietary fiber intake reduces the development of various diseases, such as inflammatory and metabolic disorders [11,12]. Therefore, SCFAs have been proposed to play a key role in the prevention and treatment of inflammatory and metabolic disorders [13,14,15]; for instance, butyrate derived from fibers improved insulin sensitivity and increased energy expenditure in a mouse model of diet-induced obesity [16].

**Figure 1:**
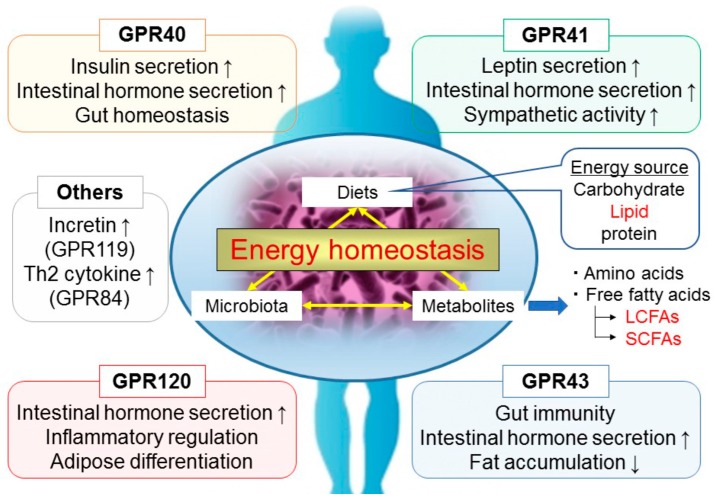
Effects of fatty acids (including SC- and LCFAs) derived from the diet by gut microbiota on the regulation of host metabolic and immune functions.

FFA receptors (FFARs) are expressed in various tissues and influence many important metabolic functions that maintain energy homeostasis. Several orphan G protein-coupled receptors (GPCRs) have been identified as FFARs; SCFAs can activate GPR41/FFAR3 and GPR43/FFAR2, while MC- and LCFAs can activate GPR40/FFAR1 and GPR120/FFAR4 (Figure 1) [17,18,19,20,21,22,23]. These FFARs have the potential as new drug targets for metabolic diseases, such as T2D and obesity.

## 2. GPR40/FFAR1

GPR40 is a receptor for MCFAs and LCFAs and is reported to bind with Gq protein. GPR40 activation increases [Ca^2+^]_i_ and activates the extracellular signal-regulated kinases (ERK1/2) signaling cascade. Gq protein is a heterotrimeric G protein subunit that activates phospholipase C (PLC) and then, intracellular Ca^2+^ release. On the other hand, it also increases cyclic (c)AMP levels via a Gs-coupled pathway [18,24]. MCFAs and LCFAs activate GPR40 at micromolar concentrations; docosahexaenoic acid (DHA) C22:6 (ω-3) and eicosapentaenoic acid (EPA) C20:5 (ω-3) are the most potent agonist of GPR40 (Table 1). Interestingly, GPR40 activation by saturated FAs depends on carbon chain length, with palmitic acid (C16) identified as potent ligands with high affinity for GPR40. GPR40 is abundantly expressed in pancreatic insulin-producing β cells and the intestine. LCFAs promote glucose-stimulated insulin secretion (GSIS) in pancreatic β cells, and the specific and acute effects of FFAs were mitigated by the loss of GPR40 function [19]. Acute and chronic effects of FFAs on insulin secretion have been investigated using *Gpr40*-deficient and pancreatic β cell-specific *Gpr40* transgenic (TG) mice [25]; the chronic effects of LCFAs on GSIS were attenuated in the *Gpr40*-deficient mice, whereas GPR40 overexpression in pancreatic β cells prevented and improved insulin sensitivity in high fat diet (HFD)-induced obese mice [26]. Furthermore, acute and chronic effects of palmitic acid on insulin sensitivity exerted in part via GPR40 have been reported [27]. The downregulation of GPR40 mRNA by exendin-4 suppressed c-Jun N-terminal kinase (JNK) and mitogen-activated protein kinase (MAPK) cascade, blocking palmitic acid-induced apoptosis in pancreatic β cells [27], while glucose and FFAs in the pancreas have been shown to regulate GPR40 protein level [28].

GPR40 expression has been identified in the intestine, immune cells, taste buds and cells of the central nervous system [28,30,31,32,33,34]. Intestinal L and K cells, which produce incretin hormones, such as glucagon-like peptide (GLP)-1 and gastric inhibitory polypeptide (GIP), as well as cholecystokinin (CCK)-producing I cells all express GPR40. Hence, LCFAs directly activate GPR40 and enhance GSIS not only by direct stimulation of insulin secretion from pancreatic β cells, but also indirectly via upregulation of GLP-1, GIP and CCK in the intestine [35,36]. In addition, in Caco-2 cells, as a human intestinal epithelial cell line, GPR40 signaling induced by a gut microbial metabolite of linoleic acid restored intestinal epithelial barrier impairments [37]. GPR40 is also expressed in bovine neutrophils [38]. The selective GPR40 agonist GW9508 can modulate bovine neutrophil responses, such as ERK1/2 phosphorylation, superoxide production, CD11b expression and matrix metalloproteinase-9 release, in an [Ca^2+^]_i_-dependent manner. In taste buds, GPR40 plays a critical role in the taste response to nutritional sensing of LCFAs [39]; the receptor is primarily expressed on the back of the tongue, including on the circumvallate and foliate papillae [32]. Although further studies are needed to clarify the function of GPR40 in taste buds, these findings suggest that GPR40 has a key role in the perception of a lipid taste. GPR40 is also widely expressed in the central nervous system, including in neurons of the cerebral cortex, hippocampus, amygdala, hypothalamus, cerebellum and spinal cord [34]. Activation of GPR40 in neuroblastoma cells induced the phosphorylation of cAMP response element-binding protein (CREB) and ERK1/2; the former may influence the secretion of brain-derived neurotrophic factor (BDNF), which plays an important role in activity-dependent regulation of synaptic structure and function [40]. TAK-875, a potent and selective small-molecule agonist of GPR40, is useful for the development of the treatment for T2D by Takeda [41].

## 3. GPR120/FFAR4

GPR120 is a receptor for MC- and LCFAs that stimulates GLP-1 secretion from L cells in the colon [42] and is activated by various ω-3 or ω-6 polyunsaturated fatty acids, including docosahexaenoic acid (DHA) C22:6 (ω-3) and eicosapentaenoic acid C20:5 (ω-3), at micromolar concentrations. However, GPR120 is strongly activated by α-linolenic acid C18:3 (ω-3) (Table 1). Although the ligands’ affinity is similar to those of GPR40, the two receptors share only 10% amino acid homology. LCFAs increased [Ca^2+^]_i_, but exerted no effects on cAMP production in human or mouse GPR120-expressing cells, indicating that GPR120 is coupled with the Gq protein family, but not with the Gi/o or Gs protein families [43]. In addition, GPR120 activates ERK1/2 and phosphoinositide 3-kinase (PI3K), although the underlying mechanisms remain unclear [44].

GPR120 is widely expressed in many tissues and cell types, such as the intestine, pancreas, adipocytes and immune cells. This suggests that GPR120 has varied roles in energy regulation and immunological homeostasis. GPR120 activation by DHA had anti-inflammatory effects in macrophages [45] associated with the suppression of Toll-like receptor signaling via the β-arrestin-2 and with the inhibition of transforming growth factor-β-activated kinase 1, which is involved in pro-inflammatory tumor necrosis factor (TNF)-α signaling. Thus, GPR120 is an ω-3 FFA receptor that improves insulin resistance and anti-diabetic effects by suppressing tissue inflammation mediated by macrophages. Moreover, stimulation of FFAs induced GLP-1 and CCK secretion in mouse enteroendocrine STC-1 cells, as a murine enteroendocrine cell line, [43,46], while *Gpr120* knockdown abolished FFA-induced effects on incretin secretion and [Ca^2+^]_i_ levels [47]. The effect of FFAs on the plasma levels of GLP-1 and insulin were examined by the administration of FFAs into the mouse colon [42]. The lipid accumulation during the adipogenesis in 3T3-L1 cells, as a murine preadipocyte cell line, induced GPR120 expression [48], and *Gpr120* knockdown in 3T3-L1 cells and the embryonic fibroblasts in *Gpr120* deficient mice inhibited the adipogenic gene expressions and prevented lipid accumulation. These findings indicate that GPR120 plays an important role in the differentiation and maturation of adipocytes. In humans, GPR120 dysfunction leads to obesity, resulting in glucose intolerance and fatty liver accompanied by decreased adipocyte differentiation and lipogenesis and enhanced hepatic lipogenesis [49]. Mutation from R (arginine) to H (histidine) at 270 amino acid sequences lacked the ability of GPR120 activation via LCFA and was significantly associated with obesity. These results provide insight into the contribution of LCFAs, which are often proposed as dietary supplements, to metabolism. Thus, GPR120 agonists could be useful for the improvement of insulin sensitivity for the treatment of T2D and other human insulin resistance [50].

## 4. GPR41/FFAR3

GPR41 is an SCFA receptor coupled with Gi/o, whose ligands include acetate (C2), propionate (C3) and butyrate (C4) [17,21]; and its ligand affinity is “propionate > butyrate > acetate” (Table 1). Recent studies have demonstrated that SCFAs produced by microbial fermentation act as signaling molecules via SCFA receptors, such as GPR41 and GPR43. Hence, gut microbiota play key roles in host physiological and pathological effects through these receptors. GPR41, which is expressed in adipose tissue, intestine and the peripheral nervous system, mediates systemic energy production by SCFAs, specifically by regulating intestinal gluconeogenesis and sympathetic activity. GPR41 expression in intestinal L cells that secrete GLP-1 and peptide YY (PYY) suggests its involvement in energy homeostasis [51]; indeed, PYY secretion from these cells is regulated by GPR41 signaling activated by gut microbiota-derived SCFAs [52]. Moreover, in primary cultured endocrine cells derived from *Gpr41*-deficient mice, Secretion of PYY and GLP-1 was reduced [53,54].

GPR41 is also abundantly expressed in the sympathetic nervous system (SNS); propionate promoted increasing energy expenditure and heart rate through GPR41 [54], effects that were not exhibited by *Gpr41*-deficient mice. Propionate is thought to control energy balance by directly regulating GPR41-mediated sympathetic activation. Under fasting conditions, β-hydroxybutyrate, which is a ketone body produced in the liver, decreased SNS activity by antagonizing GPR41, thereby reducing energy expenditure. Both SCFAs and ketone bodies reflect the nutrient status of an organism; these monocarboxylic metabolites control energy balance by directly regulating GPR41-mediated sympathetic activation. [54]. Thus, GPR41 acts as a nutritional sensor to regulate SNS activity and maintain energy balance in the body. GPR41 also enhances insulin sensitivity through the gut–brain neural circuit, which involves activation of peripheral nerve GPR41 by SCFAs produced by gut microbiota from dietary fibers [55]. GPR41 is expressed in mouse and human pancreatic β cells, as well as MIN6 cells, as a murine pancreatic β cell line, and EndoC-βH1 cells, as a human pancreatic β cell line, suggesting that it directly regulates insulin secretion [56]. Hence, SCFAs exhibit beneficial effects on the host metabolism via GPR41 that is exerted via the peripheral nervous system and hormone secretion in the gut.

## 5. GPR43/FFAR2

GPR43 is also identified as an SCFA receptor that can be activated by acetate (C2), propionate (C3) and butyrate (C4) [17,21]; and its ligand affinity is “acetate = propionate > butyrate” (Table 1). GPR43 is a Gi/o- and Gq-dual-coupled receptor that is expressed in enteroendocrine cells, such as L cells. PYY-expressing L cells in the colon were found to be immunoreactive for GPR43, while 5-hydroxytryptamine (serotonin)-positive mast cells [57]. PYY is an anorectic peptide that inhibits upper gastrointestinal tract motility and is released into the blood in response to SCFAs [58], an effect that may be mediated by activated GPR43. Additionally, GPR43 activation by SCFAs also promotes GLP-1 secretion in both colonic primary cultures and STC-1 cells [53,59]. In *Gpr43*-deficient mice, GLP-1 secretion by SCFAs reduced both *in vitro* and *in vivo*, and they have improved glucose tolerance [60]. GPR43 is also more highly expressed in the white adipose tissue of obese as compared to normal mice [60]; these investigators also reported that SCFAs inhibited lipolysis in a dose-dependent manner in 3T3-L1-derived adipocytes. These effects were dependent on GPR43 using *Gpr43*-deficient mice [61]. Moreover, obesity was induced in *Gpr43*-deficient mice by high fat diet and normal chow; however, adipose-specific *Gpr43* TG mice were lean for each diet. GPR43 activation promotes energy expenditure and permitted the preferential usage of fat through suppression of fat accumulation by inhibition of adipose tissue-specific insulin signaling. Furthermore, these strains did not show each phenotype in the germ-free mice or antibiotic-treated mice. Thus, gut microbiota are the most important source of GPR43 agonists, and GPR43 activation is closely linked to the metabolites by gut microbiota. These results showed that SCFAs as ligands for GPR43 were dependent on the presence of gut microbiota and that GPR43 regulates adipose-insulin signaling by sensing gut microbial SCFAs; thereby, these studies clearly indicated the importance of SCFAs produced by gut microbiota and their receptors [62]. This implies that the GPR43-insulin pathway is an important physiological mechanism in which metabolites are used to maintain the balance of energy in the body [63,64]. GPR43 is also expressed in pancreatic β cells and regulates insulin secretion via Ca^2+^ flux through a Gq-coupled pathway [53]. Thus, GPR43 inhibits fat accumulation in adipose tissue, promotes GLP-1 secretion in the colon and directly regulates insulin secretion in the pancreas, thereby promoting systemic insulin sensitivity. Based on these findings, GPR43 agonists may be useful for the treatment of T2D.

Many studies have investigated the role of GPR43 in regulating inflammatory responses. Stimulation of GPR43 by acetate inhibited colitis and inflammation; conversely, *Gpr43*-deficient mice showed a severe inflammatory response in colitis, arthritis and asthma, which may be related to an increase in the recruitment of immune cells [65]. On the other hand, *Gpr43*-deficient mice showed a decreased survival rate comparison with healthy mice, despite the reduction of macrophage recruitment, colonic tissue inflammation and damage in an acute colitis model [66]. This duality in the action of SCFAs, that is, anti-inflammatory and neutrophil recruitment, is a key to understanding how SCFAs and GPR43 regulate inflammation and is consistent with the roles of human monocytes and peripheral blood mononuclear cells in the inflammatory response [67]. The oral administration of SCFAs protected T-cell-transfer colitis in a GPR43-dependent manner by regulating the size and function of the colonic pool of regulatory T-cells [68]. Finally, it has been suggested that prebiotic feeding can modulate inflammatory responses in colitis, obesity, diabetes and leukemia in rodent models [69]. It can be supposed that SCFAs are produced by the fermentation of gut microbes, which may show their anti-inflammatory effects in a GPR43-dependent manner, although additional studies are needed to investigate this possibility. GLPG0974 is an orally-available small GPR43 inhibitor, and was developed by Galapagos. This antagonist might have potential for the treatment of inflammatory bowel disease (NCT01829321).

## 6. Other GPCRs

Whereas LCFAs and SCFAs derived from the diet act as signaling molecules through GPCRs, GPR119 (a receptor for fatty acid amides and monoacylglycerol) and GPR84 (a receptor for MCFAs) also have physiological functions as nutritional receptors.

GPR119 is predominantly expressed in human and mouse enteroendocrine and pancreatic β cells. Oleoylethanolamide is an endogenous ligand for GPR119 and a peripherally-acting agent that reduces food intake and body weight gain in rat feeding models [70]. In addition, ligands, such as 2-monoacylglycerols, 2-oleoylglycerol, 2-palmitoylglycerol and 2-linoleoylglycerol, can activate GPR119 (Table 2) [71,72,73]. Thus, metabolic disorders can potentially be treated by inducing GPR119 activation and stimulating both GLP-1 and insulin secretion. GPR119 induces incretins, such as GLP-1 and GIP, in enteroendocrine cells [74]. In pancreatic β cells, GPR119 was shown to induce GSIS via cAMP production, suggesting that the receptor functions through activation of Gαs [75]. GPR119-mediated insulin secretion from the pancreas is glucose dependent, whereas GPR119-mediated GLP-1 secretion from enteroendocrine cells is thought to be glucose independent [76].

GPR84 is an MCFA receptor that is strongly activated by undecanoic acid (C11) and lauric acid (C12) (Table 3) [77]. GPR84 is associated with the pertussis toxin-sensitive Gi/o pathway and is mainly expressed in bone marrow and to a lesser degree in peripheral leukocytes and lungs [77,78]. GPR84 is highly expressed in monocytes and neutrophils upon lipopolysaccharide stimulation. Additionally, RAW264.7 cells, a mouse monocyte/macrophage cell line, produce the IL-12 p40 subunit upon MCFA stimulation [77]. Inducing and maintaining T helper (Th)1 response is a critical role of cell-mediated immunity, while inhibiting Th2 response is important for antibody-mediated immunity [79]. *Gpr84*-deficient mice exhibited an enhanced Th2 response, reflecting increased Th2 cytokine production [80]. Moreover, GPR84 was upregulated in a 3T3-L1 adipogenesis model and in the adipose tissue of obese patients and mice fed a HFD [80]. GPR84 in adipose tissue aggravates the pathogenesis of obesity and T2D via TNF-α released by infiltrating macrophages. Thus, GPR84 plays a pivotal role in metabolism and immune responses and may mediate crosstalk between immune cells and non-immune cells, such as adipocytes.

## 7. Conclusions

Host metabolic and immunological disorders have been linked to diet and gut microbiota composition. Many researchers have reported that FFARs are expressed in various tissues and cells as nutritional sensors and regulate both metabolic and inflammatory responses (Figure 1). In the future, FFAR signaling in diverse metabolic processes will provide new insights for nutritional science and drug discovery. Since the development of metabolic disorders, such as obesity and T2D, is caused by inflammation, the anti-inflammatory effects of FFARs are expected to be an important therapeutic target for the treatment of these diseases. It is well established that gut microbiota composition closely influences host energy metabolism and inflammatory responses; the discovery of FFARs, including GPR41, GPR43, GPR40, GPR84, GPR119 and GPR120, as receptors for gut microbial metabolites provides a molecular link that can explain these interactions. In conclusion, although further investigation of the relationship between FFARs and health is needed to clarify the underlying mechanisms, it might have an important role in the treatment of inflammatory and metabolic disorders and the development of therapeutic drugs.

## Figures and Tables

**Table 1:** Affinity of free fatty acids for GPR41, GPR43, GPR40 and GPR120 [29].

Ligand	EC_50_ of Ligand Affinity (μM)			
	GPR41/FFAR3	GPR43/FFAR2	GPR40/FFAR1	GPR120/FFAR4
**Saturated fatty acids**				
acetic acid (C2:0)	>1000 ^(b–d)^	35–431 ^(b–d)^		
propionic acid (C3:0)	6–127 ^(b–d)^	14–290 ^(b–d)^		
butyric acid (C4:0)	42–158 ^(b–d)^	28–371 ^(b–d)^		
valeric acid (C5:0)	42–142 ^(b–d)^	>1000 ^(b–d)^		
caproic acid (C6:0)	102–134 ^(a,c,d)^		46 ^(a,c,d)^	
caprylate (C8:0)			38 ^(a)^	
capric acid (C10:0)			14–43 ^(a,d)^	
lauric acid (C12:0)			6–12 ^(a,d)^	
myristic acid (C14:0)			8–14 ^(a,d)^	30 ^(a,d)^
palmitic acid (C16:0)			5–7 ^(a,d)^	52 ^(a,d)^
stearic acid (C18:0)			17 ^(a)^	18 ^(a)^
**Monounsaturated fatty acids**				
palmitoleic acid (C16:1, n-7)			14 ^(a)^	0.7–3 ^(a)^
oleic acid (C18:1, n-9)			2–40 ^(a,d)^	31 ^(a,d)^
**ω-3 fatty acids**				
α-linolenic acid (C18:3, n-3)			2–13 ^(a,d)^	0.5 ^(a,d)^
cis-11,14,17-eicosatrienoic acid (C20:3, n-3)			11 ^(a)^	1 ^(a)^
cis-5,8,11,14,17-eicosapentaenoic acid (C20:5, n-3)			2–7 ^(a,d)^	2–3 ^(a,d)^
docosahexaenoic acid (22:6, n-3)			1–4 ^(a,d)^	4 ^(a,d)^
**ω -6 fatty acids**				
linoleic acid (C18:2, n-6)			2–10 ^(a,d)^	1 ^(a,d)^
γ-linolenic acid (C18:3, n-6)			5–9 ^(a,d)^	1 ^(a,d)^
dihomo-γ-linolenic acid (C20:4, n-6)			7 ^(a)^	14 ^(a)^
arachidonic acid (C20:4, n-6)			2–12 ^(a,d)^	
docosatetraenoic acid (C22:4, n-6)			13 ^(a)^	16 ^(a)^

Measured as induction of [Ca^2+^]_i_^(a)^ and GTPγs ^(b)^ in each FFARs-transfected HEK293 cells, or cAMP ^(c)^ and [Ca^2+^]_i_^(d)^ in each FFARs-transfected CHO cells.

**Table 2:** Affinity of representative compounds including fatty acids for GPR119 [74].

Ligand	EC_50_ (μM)
all trans-retinoic acid	no response
C18:1 LPC	no response
oleoylethanolamide (OEA)	4.4
Z-capsaicin	no response
olvanil	7.8
*N*-oleoyldopamine (OLDA)	3.2
(*R*)-*N*-oleoyltyrosinol	0.5
(*S*)-*N*-oleoyltyrosinol	0.7
*N*-oleoyltryosine	no response
*N*-arachidonyldopamine (NADA)	no response

Measured as induction of cAMP in GPR119-transfected HEK293 cells.

**Table 3:** Affinity of various fatty acids for GPR84 [77].

Ligand	EC_50_ (μM)
formic acid (C1:0)	no response
acetic acid (C2:0)	no response
propionic acid (C3:0)	no response
butyric acid (C4:0)	no response
caproic acid (C6:0)	no response
heptanoic acid (C7:0)	no response
caprylic acid (C8:0)	no response
nonanoic acid (C9:0)	52.3 ± 5.6
capric acid (C10:0)	4.5 ± 0.3
undecanoic acid (C11:0)	7.7 ± 0.1
lauric acid (C12:0)	8.8 ± 0.2
tridecanoic acid (C13:0)	24.8 ± 1.1
myristic acid (C14:0)	93.2 ± 11.0
pentadeconoic acid (C15:0)	no response
palmitic acid (C16:0)	no response
heptadecanoic acid (C17:0)	no response
stearic acid (C18:0)	no response
arachidic acid (C20:0)	no response
heneicosanoic acid (C21:0)	no response
behenic acid (C22:0)	no response
palmitoleic acid (C16:1)	no response
oleic acid (C18:1)	no response
elaidic acid (C18:1)	no response
linoleic acid (C18:2)	no response
α-linolenic acid (C18:3)	no response
γ-linolenic acid (C18:3)	no response
cis-11,14,17-eicosatrienoic acid (C20:3)	no response
arachidonic acid (C20:4)	no response
cis-5,8,11,14,17-eicosapentaenoic acid (C20:5)	no response
docosahexaenoic acid (C22:6)	no response

Measured as induction of cAMP in GPR84-transfected CHO cells.
